# Improving the evaluation of cardiac function in rats at 7T with denoising filters: a comparison study

**DOI:** 10.1186/s12880-017-0236-2

**Published:** 2017-12-19

**Authors:** Benoit Tricot, Maxime Descoteaux, Matthieu Dumont, Frederic Chagnon, Luc Tremblay, André Carpentier, Olivier Lesur, Martin Lepage, Alain Lalande

**Affiliations:** 1 Centre d’Imagerie Moléculaire de Sherbrooke, CHUS - Hôpital Fleurimont, Sherbrooke, Canada; 2 Center for Systems Biology, Massachusetts General Hospital and Harvard Medical School, Boston, USA; 3 Département d’Informatique, Université de Sherbrooke, Sherbrooke, Canada; 4 Soins Intensifs Médicaux, CHUS - Hôpital Fleurimont, Sherbrooke, Canada; 5 Département de Médecine, CHUS - Hôpital Fleurimont, Sherbrooke, Canada; 6 Le2I, Faculté de Médecine, Université Bourgogne Franche-Comté, 7 Bld Jeanne d’Arc, 21079 Dijon Cedex, BP 87900 France

**Keywords:** Small animal, Non-local means filtering, Cine-MRI, Denoising

## Abstract

**Background:**

We investigate the use of different denoising filters on low signal-to-noise ratio cardiac images of the rat heart acquired with a birdcage volume coil at 7T. Accuracy and variability of cardiac function parameters were measured from manual segmentation of rat heart images with and without filtering.

**Methods:**

Ten rats were studied using a 7T Varian system. End-diastolic and end-systolic volumes, ejection fraction and left ventricle mass (LVM) were calculated from manual segmentation by two experts on cine-FLASH short-axis slices covering the left ventricle. Series were denoised with an anisotropic diffusion filter, a whole variation regularization or an optimized Rician non-local means (ORNLM) filtering technique. The effect of the different filters was evaluated by the calculation of signal-to-noise (SNR) and contrast-to-noise (CNR) ratios, followed by a study of intra- and inter-expert variability of the measurement of physiological parameters. The calculated LVM was compared to the LVM obtained by weighing the heart ex vivo.

**Results:**

The SNR and the CNR increased after application of the different filters. The performance of the ORNLM filter was superior for all the parameters of the cardiac function, as judged from the inter- and intra-observer variabilities. Moreover, this filtering technique resulted in the lowest variability in the LVM evaluation.

**Conclusions:**

In cardiac MRI of rats, filtering is an interesting alternative that yields better contrast between myocardium and surrounding tissues and the ORNLM filter provided the largest improvements.

## Background

Magnetic Resonance Imaging (MRI) is a valuable tool for the detection of cardiovascular diseases. Owing to its fair temporal and high spatial resolutions, MRI is now an established and reliable technique for the assessment of cardiac structure, function, perfusion, and it can assess myocardial viability [1]. Animal models, and particularly transgenic rats and mice, are increasingly used for the study of genes and biological mechanisms involved in heart diseases [2]. MRI has become the gold-standard for the non-invasive examination of these models. This is despite challenges imposed by an elevated heart rate (up to 600 beats per minute for the mouse) and small heart [3]. High-quality heart images of animal models are typically acquired with dedicated high-field MRI systems, respiratory and cardiac gating strategies as well as optimized pulse sequences. Dedicated multi-element cardiac coil arrays can further increase the signal-to-noise ratio (SNR) in images of the rat heart [4], and this is particularly relevant for fast and kinetic imaging. However, these coils are not widely available and their cost is relatively high. Birdcage volume coils provide a lower SNR but they have been more popular and more widely used because of their versatility and their superior radio-frequency homogeneity.

In our study, we investigated the effect of three filtering techniques applied to low-SNR cine images of the rat heart acquired with a birdcage volume coil. In particular, we assessed their effect on the accuracy and the variability of cardiac function parameters obtained from these images. The filtering techniques investigated were an anisotropic diffusion filter routinely used in image processing, a total variation regularization filter and an optimized Rician non-local means (ORNLM) filter [5]. Intra- and inter-observer variabilities were assessed for the following parameters: end-diastolic volume (EDV), end-systolic volume (ESV), ejection fraction (EF) and left ventricle mass (LVM). The LVM obtained after image analysis was also compared to the LVM measured ex vivo [6]. The main goal was to determine whether evaluation of the cardiac function with a birdcage volume coil could be improved by applying filtering technique as post-processing.

## Methods

### MRI acquisition

Ten (*n* = 10) male and female Fischer rats of varying age (7–14 weeks) and varying weights (roughly from 100 to 270 g) were imaged using a 7T Varian scanner, with a 63 mm-diameter volume coil. Series of short-axis cine images covering the left ventricle were obtained with an ECG and respiratory-gated cine-FLASH sequence (TR: 80% of the R-R interval: typically around 166–180 ms; TE: 2.10 ms; flip angle: 20^o^; matrix size: 256 × 256; in plane resolution: 195 × 195 μm^2^; 15 slices; slice thickness: 1 mm; 16 frames covering the entire cardiac cycle) [7]. The acquisition time for one short-axis plane was between 78 s and 138 s depending on heart and respiration rates. The total acquisition time was between 25 and 44 min depending on the number of slices that were required to cover the entire left ventricle. The protocol was approved by the institutional animal ethics board, which follows the guidelines issued by the Canadian Council on Animal Care. Animals were anesthetized with isoflurane during imaging (1.5% in air). After imaging, they were euthanized by CO2 inhalation and their left ventricles were excised and weighed.

### Image processing

Three denoising filters were tested on the entire data set, which was split in 16 different 3D volumes (one for each phase of the cardiac cycle). Each 3D volume was made up of the totality of the short-axis slices covering the entire left ventricle for one specific phase of the cardiac cycle. The three filtering techniques were: i) anisotropic diffusion filtering [8], ii) total variation regularization [9] and iii) non-local means with optimized Rician noise bias correction (ORNLM) [5]. Anisotropic filtering is based on the anisotropic diffusion heat equation [8]. This is an edge-preserving filter in that smoothing occurs along structures and not across them, as opposed to isotropic or linear diffusion, which are equivalent to Gaussian smoothing. The total variation regularization attempts to promote the piecewise constant nature of images by enhancing edges and by smoothing across homogeneous and flat-intensity regions. It is also strongly based on the gradients of the images. Finally, due to the Rician noise nature of cardiac MR images, the last technique that we tested was the non-local means approach with Rician bias correction [10] implemented in Dipy [11]. For ORNLM, each 3D volume was processed using non-local means filtering with automatic noise standard deviation estimation of the background, search volume of 1331 neighbors and neighborhood size of 26 neighbors.

The three denoising filters were implemented in an in-house built software using third party libraries. This software was written in Python and was run on a Windows 7 platform. The ORLNM implementation was taken from Dipy (http://nipy.org/dipy/). For the anisotropic diffusion filter, the 3D implementation of the algorithm was taken from SimpleITK (http://www.simpleitk.org/). Finally the total variation regularization was taken from scikit (http://scikit-image.org/).

### Image analysis

The SNR and the contrast-to-noise ratio (CNR) were calculated on a mid-ventricular short-axis slice at end-diastole for each data set. Regions of interest (ROIs) were drawn in the blood pool, in the myocardium and in the background of the image for noise estimation. The same regions of interest were used with three different filtering strategies and the background area was located outside the animal in the bottom right of the image. As the SNR is higher than 4, the Rician noise distribution can be approximated by a Gaussian distribution [12], and then the SNR of the blood and the myocardium were calculated as the ratio between the mean signal intensity in the corresponding ROI and the standard deviation of the signal in the background of the image. The CNR was calculated as the difference between the SNR of the blood and the SNR of the myocardium.

Cine images were manually segmented by two experienced observers using the Segment software (http://medviso.com/products/segment/) [13]. The segmentation was done twice by the same observer (observer 1) over a two-month period, and once by the second observer (observer 2). The first segmentation of observer 1 was considered for the comparison between the two observers. End-diastolic and end-systolic volumes (EDV, ESV), ejection fraction (EF) and left ventricle mass (LVM) were calculated (defined as the myocardial volume multiplied by 1.05 g.cm^−3^). A two-tailed paired Student’s t-test was used to determine any significant differences between measurements from different segmentations. The correlation coefficients were calculated between the two observers and between the two measurements by observer 1 for each parameter. Agreement for intra and inter-observer observations was tested by calculating the difference between the two measures, taking the first measure of observer 1 as the reference. There was a high variation in the heart weights ex vivo such that results are presented in terms of an absolute but also relative differences (%). In this latter case, for each parameter, the difference between the two measurements was normalized to their average. The results are expressed in absolute values (respectively in percentage) as mean ± standard deviation. Finally, the LVM obtained from the images was compared to the LVM calculated from the ex vivo heart weight, which was used as the gold standard. The results were calculated as a percentage difference from the ex vivo value and are displayed as mean ± standard deviation.

## Results

**Fig. 1:**
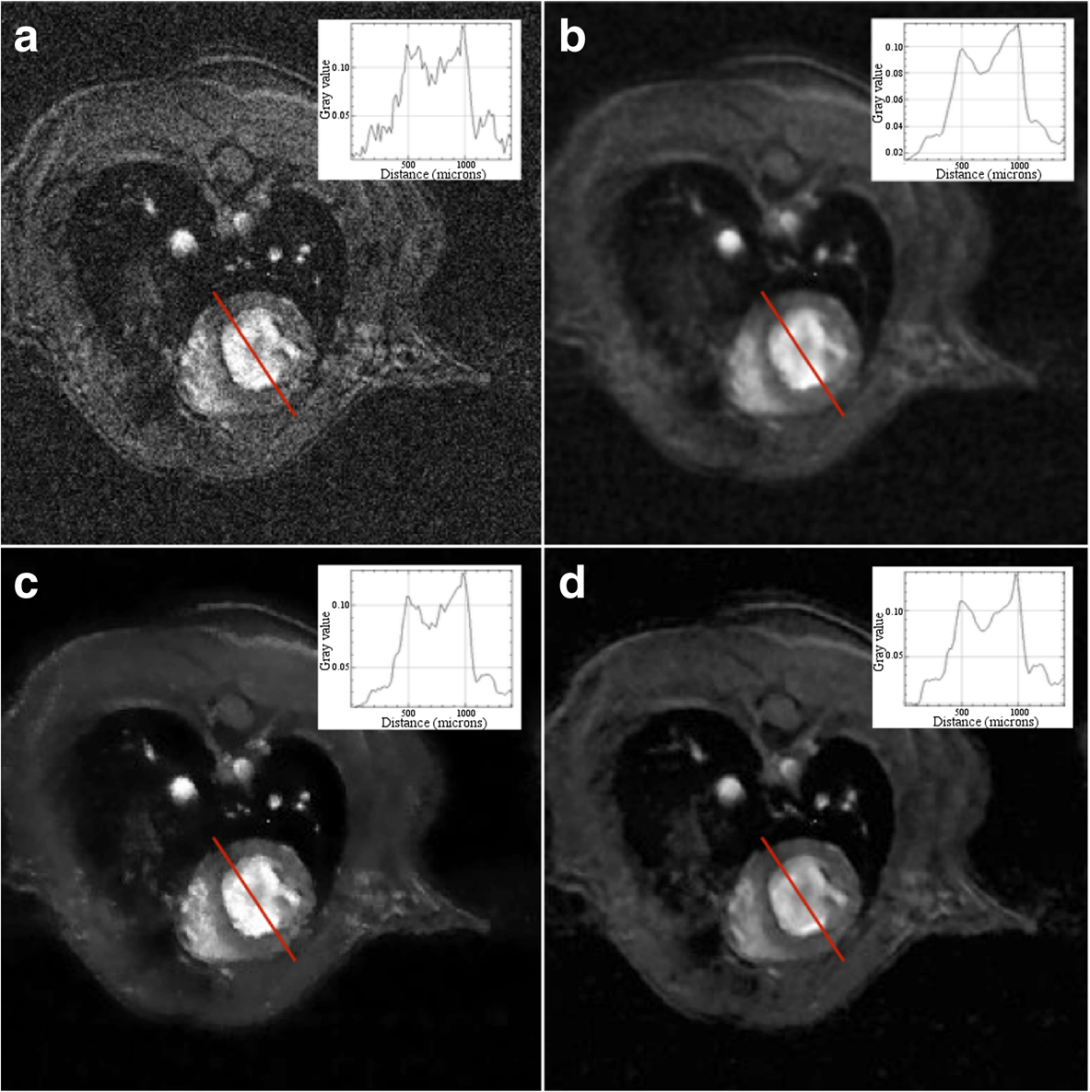
Example of image filtering on a short axis slice. For each image, the plot displays the signal along the corresponding red line located at the level of the left ventricle. With FLASH sequence, the blood appears in white and the myocardium in grey. **a**) raw image (no filter), **b**) anisotropic filter, **c**) total variation filter and **d**) ORNLM filter

Figure 1 shows typical results of the post-processing with the different filtering techniques on a short-axis slice. Visually, the noise is reduced in the filtered images than in the raw image. The mean total time for processing a data set is roughly equal to 0.72 min for the anisotropic filter, to 0.63 min for the total variation regularization and to 10 min for ORNLM. Table 1 shows the mean SNR of the blood and the myocardium, as well as the CNR between the blood and the myocardium for the raw images and the filtered images. The mean SNR increases at least 8-fold with all filters compared to the raw images. The total-variation filter resulted in the highest mean SNR but also in a high variability as shown by the high value of the standard deviation. The ORNLM filter yielded the second highest mean SNR with a lower standard deviation. The anisotropic diffusion filter yielded the smallest improvement in SNR and CNR but the standard deviations were also the lowest.

**Table 1:** Image quality with the different filtering techniques

	SNR blood	SNR myocardium	CNR
No filter	12 ± 2	5 ± 1	6 ± 2
Anisotropic filter	82 ± 15	39 ± 7	43 ± 11
Total variation filter	192 ± 133	89 ± 60	103 ± 76
Optimized Rician Non-local Means filter	110 ± 74	46 ± 35	64 ± 41

Mean signal to noise ratio (SNR) of the blood and of the myocardium and contrast to noise ratio (CNR) between the blood and the myocardium on raw and filtered images

The mean values of the EDV and ESV measured with the different filters by observer 1 ranged from 382 μL to 888 μL and from 104 μL to 261 μL, respectively. The EF values varied between 67% and 80% and the LVM values between 453 mg and 1010 mg. Even though the rats were healthy, we selected a large range of rat size in order to test our approach on a wide range of heart size, which explain the variation in cardiac volumes and masses between rats.

**Fig. 2:**
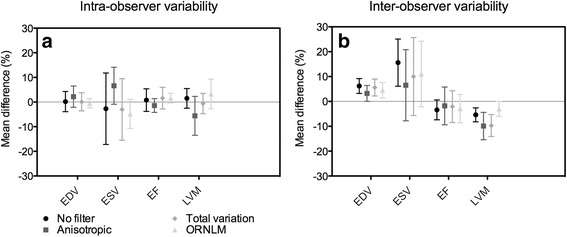
**a**) Intra and **b**) inter-observer variabilities for the calculation of the different parameters. The graphics display the relative mean difference ± standard deviation

The results of inter and intra-observer studies are summarized in Tables 2 and 3, and Fig. 2.

**Table 2:** Intra observer variation study for all parameters on raw and filtered images

	Difference	Difference (%)	r
No filter			
EDV (μL)	0.5 ± 16.3	0.2 ± 4.1	0.995
ESV (μL)	−2.4 ± 17.2	−2.7 ± 14.5	0.939
EF (%)	0.7 ± 3.2	0.8 ± 4.6	0.725
LVM (mg)	13.2 ± 28.7	1.5 ± 4.0	0.994
Anisotropic filter			
EDV (μL)	7.6 ± 22.0	2.2 ± 4.3	0.995
ESV (μL)	8.4 ± 13.2	6.6 ± 7.5	0.977
EF (%)	−1.3 ± 1.6	−1.4 ± 2.8	0.950
LVM (mg)	−35.1 ± 49.9	−5.6 ± 7.9	0.972
Total variation filter			
EDV (μL)	0.8 ± 22.2	0.1 ± 3.7	0.991
ESV (μL)	−4.7 ± 16.3	−3.0 ± 12.5	0.955
EF (%)	1.0 ± 3.1	1.6 ± 4.4	0.870
LVM (mg)	−0.4 ± 31.2	−0.6 ± 4.1	0.986
Optimized Rician non-Local Means filter			
EDV (μL)	−0.3 ± 7.7	−0.5 ± 1.9	0.999
ESV (μL)	−4.8 ± 6.9*	−4.8 ± 5.9*	0.989
EF (%)	1.1 ± 1.5*	1.6 ± 2.0*	0.934
LVM (mg)	12.6 ± 30.5	3.3 ± 6.0	0.983

Correlation coefficient (r) and evaluation of the absolute and relative mean difference (%). EDV: End diastolic volume; ESV: End systolic volume; EF: Ejection fraction; LVM: Left ventricular mass. *p⩽ 0.05

**Table 3:** Inter observer variation study for all parameters on raw and filtered images

	Difference	Difference (%)	r
No filter			
EDV (μL)	31.7 ± 13.0	6.2 ± 3.5***	0.998
ESV (μL)	23.5 ± 21.6	14.4 ± 14.0**	0.926
EF (%)	−2.3 ± 3.9	−3.3 ± 5.6*	0.544
LVM (mg)	−32.8 ± 23.8	−5.2 ± 3.9**	0.993
Anisotropic filter			
EDV (μL)	21.3 ± 16.1	4.3 ± 3.6**	0.996
ESV (μL)	13.3 ± 22.1	9.9 ± 14.9	0.909
EF (%)	−1.7 ± 4.9	−2.4 ± 7.6	0.692
LVM (mg)	−81.8 ± 48.9	−12.5 ± 8.4***	0.971
Total variation filter			
EDV (μL)	28.7 ± 19.2	5.8 ± 4.3**	0.994
ESV (μL)	11.4 ± 24.6	8.6 ± 18.1	0.856
EF (%)	−0.9 ± 4.4	−1.3 ± 6.4	0.706
LVM (mg)	−63.3 ± 30.1	−10.0 ± 5.5***	0.987
Optimized Rician non-Local Means filter			
EDV (μL)	26.9 ± 19.1	4.7 ± 3.2**	0.997
ESV (μL)	19.5 ± 20.2	13.5 ± 13.9*	0.904
EF (%)	−2.4 ± 3.7	−3.2 ± 6.3	0.764
LVM (mg)	−29.2 ± 27.0	−4.6 ± 3.9**	0.991

Correlation coefficient (r) and evaluation of the absolute and relative mean difference (%). EDV: End diastolic volume; ESV: End systolic volume; EF: Ejection fraction; LVM: Left ventricular mass. *p⩽ 0.05, **p⩽ 0.01, ***p⩽ 0.001

For the intra-observer variability, there was an excellent coefficient correlation (*r* > 0.934) between the two measurements by observer 1 for all the parameters, except for the EF derived from raw images (*r* = 0.725) and to a lesser degree for the EF with the total variation filter (*r* = 0.870). The agreement analysis revealed that the mean difference was less than 5% for all the parameters with the different filters, except for ESV and LVM with the anisotropic filter (−6.6% and 5.6%, respectively).

For the inter-observer variability, there was also an excellent correlation (*r* > 0.904) between the two observers for all the parameters with the exception of EF, regardless of the filtering method used (r values ranging from 0.544 to 0.764), and of ESV with total variation filter. The ORLNM filter yielded the best correlation between the two observers for EF (*r* = 0.764). On the contrary, the unfiltered images yielded the lower correlation. The agreement analysis indicated a bias between the two observers (mean difference > 8.5%) for the evaluation of the systolic volume (ESV), regardless of the type of images being used. However, all the other parameters (EDV, EF and LVM) had a mean difference of less than 5% between the two observers only after application of the ORNLM filter. There were significant inter-observer differences (*p* < 0.05) in all parameters derived from raw images. After filtering, the differences decreased and became non-significant for some parameters, particularly for EF. For the intra-observer comparison, no significant difference was found between the two measurements, except for ESV and EF calculated after application of the ORNLM filter with *p*-values just below 0.05.

**Fig. 3:**
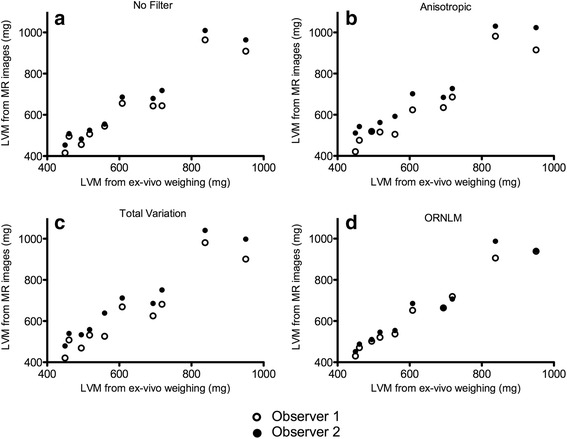
LVM obtained from the images and by weighing the LV ex-vivo for the different approaches. Comparison of the values obtained by weighing the LV ex-vivo (Fischer rats, weights from roughly 100 to 270 g) and from images with the different following approaches: **a**) no filter, **b**) anisotropic filter, **c**) total variation filter and **d**) ORNLM filter

The LVM values obtained from the images and the LVM obtained by weighing the left ventricle ex vivo are reported in Table 4 and displayed in Fig. 3. The correlation coefficient was excellent in all cases (*r* > 0.943). The mean percentage error was lower than 5% for observer 2 regardless of the type of images, whereas it became higher than 5% for observer 1 when analyzing the images filtered with the anisotropic diffusion filter and the total variation filter (8.3% and 8.8% respectively). The lowest variability (i.e. standard deviation) was obtained with the ORNLM filter for both observers (5.1% for observer 1 and 3.8% for observer 2).

**Table 4:** Correlation coefficient (r) and agreement (mean of difference) studies between in-vivo (from raw or filtered images) ex-vivo left ventricular weight estimation

	Observer 1		Observer 2	
	Difference (%)	r	Difference (%)	r
No filter	2.4 ± 5.4	0.961	−2.9 ± 6.7	0.947
Anisotropic filter	8.3 ± 6.4	0.960	−2.5 ± 5.3	0.951
Total variation filter	8.8 ± 6.2	0.960	−1.6 ± 7.5	0.943
Optimized Rician Non-Local Means filter	2.3 ± 5.1	0.960	−0.2 ± 3.8	0.986

## Discussion

Dedicated multi-element cardiac radio-frequency coils can provide high quality images of the rat heart. When this type of coil is not available, an alternative is to use a birdcage volume coil and to increase the number of acquisitions (averages) in order to increase the SNR. This solution is limited as it increases the acquisition time and the duration of anesthesia.

Our results suggest that ORNLM filtering is a reliable tool to compensate for the low SNR of cardiac images acquired with a birdcage volume coil. Although the processing time is a little longer than with the other filtering methods, it remains relatively fast. All filters tested in this study were found to improve significantly the SNR and the CNR. The total variation regularization filter yielded the largest improvement. However, the contours of the cardiac tissue were better delineated with the ORLNM filter, especially at the interface between the myocardium and the chest cavity wall. This visual observation is confirmed by the inter-observer study, where the differences in the LVM found by the two observers were lower than with the raw images, whereas these differences increased with the anisotropic filter and total variation regularization. In addition, the error and the variability in the evaluation of the cardiac function was minimized with the ORLNM filter. Moreover, the agreement between the LVM determined from ORNLM-filtered images and the LVM calculated from the ex vivo heart weight was the highest (according to the lowest values of difference in the Table 4). The higher standard deviation in SNR with filtering was due to differences in signal intensity between acquisitions and sometimes the presence of artefacts in the background areas, and these two effects were amplified with filtering.

The differences between the two observers for the end-systolic volume were higher than for any other parameter. This could be explained by the contraction of the papillary muscles, as well as the relatively high EF of healthy rats, which makes it harder to distinguish the left ventricle cavity at end systole. Despite this, the differences in EF between the two observers were non-significant after filtering the images. This is an important observation as EF is arguably the most important parameter when evaluating cardiac function. ORLNM filtering also provided convincing results in the intra-observer study, reducing the variability in the measurements made by the same observer. However, the differences between the two measurements by the same observer were found to be significant for EF and ESV with this filter, despite low mean differences and standard deviations. We note that the coefficient of correlation is relatively low for EF, due to the low number of rats, and we expect that these values would increase if the number of rats increased. However, in our opinion, the ranking will stay the same and then the ORNLM filter should provide the best results even with more data.

It should be noted that unfiltered images perform reasonably well with respect to the cardiac function measurements as well as for the left ventricular weight estimation. However, the important increase of SNR and CNR using filtering and the significant differences between observers without filtering for the calculation of the EF (with low correlation coefficient) demonstrate the necessity to perform filtering (and in particular ORLNM filtering).

A limitation of this study concerns the comparison between in vivo and ex vivo data for the measurement of LVM. Although weighing the left ventricle ex vivo is the best way to have absolute comparison, the different physiological conditions (between in vivo and ex vivo) and the excision of the heart can induce errors in measurements which could decrease the correlation and the agreement between ex vivo and in vivo results. Moreover, the choice of acquiring 16 phases of the cardiac cycle was made as a trade-off between image quality and acquisition time, and this sample rate may not be high enough to capture cleanly the systolic motion. However, it is unclear whether more phases would have improved the quality of the manual segmentation. A limitation of the results is that reproducibility was evaluated on only one set of in vivo image acquisition, thus evaluating the influence of filtering on the variation due to segmentation only. Future studies are needed to fully evaluate reproducibility on two different sets of in vivo image acquisitions. Finally, this study should be extended to other small animals such as mice.

## Conclusion

In conclusion, multi-element cardiac coil arrays are not always available for cardiac MRI in small animals. Filtering images after acquisition could be an alternative to the use of these coils. ORNLM filtering was superior to an anisotropic filter and to a total variation filter and could potentially also improve images acquired with a phased array coil.

## Abbreviations

CNR: Contrast-to-noise ratio
ECG: Electrocardiogram
EDV: End-diastolic volume
EF: Ejection fraction
ESV: End-systolic volume
FLASH: Fast low angle shot
LVM: Left ventricle mass
MRI: Magnetic Resonance Imaging
ORNLM: Optimized Rician non-local means filter
ROI: Region of interest
SNR: Signal-to-noise ratio
TE: Echo time
TR: Repetition time
